# Integrating Cytogenetics and Population Genomics: Allopatry and Neo-Sex Chromosomes May Have Shaped the Genetic Divergence in the *Erythrinus erythrinus* Species Complex (Teleostei, Characiformes)

**DOI:** 10.3390/biology11020315

**Published:** 2022-02-16

**Authors:** Fernando H. S. de Souza, Francisco de M. C. Sassi, Pedro H. N. Ferreira, Luiz A. C. Bertollo, Tariq Ezaz, Thomas Liehr, Manolo F. Perez, Marcelo B. Cioffi

**Affiliations:** 1 Laboratório de Citogenética de Peixes, Departamento de Genética e Evolução, Universidade Federal de São Carlos, São Carlos 13565-905, SP, Brazil; fernando_hsouza@outlook.com.br (F.H.S.d.S.); fmcsassi@estudante.ufscar.br (F.d.M.C.S.); pedro_henriquenarcisoferreira@hotmail.com (P.H.N.F.); bertollo@ufscar.br (L.A.C.B.); manolofperez@gmail.com (M.F.P.); mbcioffi@ufscar.br (M.B.C.); 2 Institute for Applied Ecology, University of Canberra, Canberra 2617, Australia; tariq.ezaz@canberra.edu.au; 3 Institute of Human Genetics, University Hospital Jena, 07747 Jena, Germany

**Keywords:** DArTseq, evolution, speciation, Neotropical fish, karyotype, population structure

## Abstract

**Simple Summary:**

Fish present astonishing diversity, comprising more species than the combined total of all other vertebrates. Here, we integrated cytogenetic and genomic data to investigate how the evolution of multiple sex chromosomes together with allopatry is linked to genetic diversity and speciation in the fish species *Erythrinus erythrinus*. We hypothesized that the presence of multiple sex chromosomes has contributed to the genetic differentiation of populations, which could have potentially accelerated speciation.

**Abstract:**

Diversity found in Neotropical freshwater fish is remarkable. It can even hinder a proper delimitation of many species, with the wolf fish *Erythrinus erythrinus* (Teleostei, Characiformes) being a notable example. This nominal species shows remarkable intra-specific variation, with extensive karyotype diversity found among populations in terms of different diploid chromosome numbers (2*n*), karyotype compositions and sex chromosome systems. Here, we analyzed three distinct populations (one of them cytogenetically investigated for the first time) that differed in terms of their chromosomal features (termed karyomorphs) and by the presence or absence of heteromorphic sex chromosomes. We combined cytogenetics with genomic approaches to investigate how the evolution of multiple sex chromosomes together with allopatry is linked to genetic diversity and speciation. The results indicated the presence of high genetic differentiation among populations both from cytogenetic and genomic aspects, with long-distance allopatry potentially being the main agent of genetic divergence. One population showed a neo-X_1_X_2_Y sexual chromosome system and we hypothesize that this system is associated with enhanced inter-population genetic differentiation which could have potentially accelerated speciation compared to the effect of allopatry alone.

## 1. Introduction

The biodiversity of Neotropical freshwater fish is remarkable, as they comprise at least 5200 species [1]. The Erythrinidae family is widely distributed in this region, with a marked preference for lentic environments, such as small and large rivers and lagoons [2]. The family currently contains 18 species that have been described, within three genera: *Hoplias* (13), *Hoplerythrinus* (3), and *Erythrinus* (2) [2]. However, some authors have suggested a need for a taxonomic revision [3,4], as all genera display several undescribed morphotypes which are currently included in some nominal species [5].

Such cryptic diversity is at least partially explained by the high karyotypic diversity found in some species, such as *E. erythrinus* and *H. malabaricus*, in which many studies have shown the presence of intraspecific variants, with extensive differences observed in the diploid chromosome numbers (2*n*), karyotype compositions and different sex chromosome systems among populations [4]. Four different karyomorphs are found in *E. erythrinus* (classified as A, B, C and D) with a prevalence of acrocentric chromosomes which distinguish the species karyotypes from those of other erythrinids [3,6]. While karyomorph A presents 2*n* = 54 chromosomes in both males and females, without the presence of differentiated sex chromosomes, karyomorphs B, C and D display a multiple ♀X_1_X_1_X_2_X_2_/♂X_1_X_2_Y sex chromosome system, in which males and females have 2*n* = 51 and 2*n* = 52, respectively [3,7].

Even though allopatry is generally considered the main factor for the emergence of reproductive isolation and the reduction of gene flow that culminates in the speciation process [8], the impact of chromosome rearrangements in speciation has also been widely discussed since the work of Haldane [9]. In animals, most postzygotic isolation cases are caused by genetic incompatibilities, among which chromosomal rearrangements and (the origin of) sex chromosomes may play a fundamental role [10,11,12,13]. Chromosomal rearrangements have the potential to limit introgression, thus facilitating the origin and maintenance of reproductive isolation through recombination suppression [14,15]. In addition, chromosome rearrangements involving sex chromosomes may lead to the formation of new linkage groups between genes originally found on different chromosomes, which may accelerate the accumulation of genetic incompatibilities among groups of individuals [16]. These rearrangements increase the quantity of sex-linked genes and accelerate the accumulation of genetic incompatibilities between populations [16]. When compared, species that present heteromorphic sex chromosomes show stronger reproductive isolation than species in which they are absent [17,18]. In particular, in contrast to the gradual process of recombination suppression that occurs in simple sex chromosome systems, multiple sex chromosomes can immediately suppress recombination in regions that are close to the breakpoints [19,20]. These systems generally arise after translocation events or fusion between an autosome and an existing sex chromosome. In meiotic segregation, a meiotic multivalent is formed that may cause problems in segregation, generating unbalanced gametes, thus leading to a postzygotic barrier (reviewed in [21]). Therefore, the formation of a multiple sex chromosome system can promote fast recombination suppression, act as a postzygotic barrier and contribute to reproductive isolation.

Recently, high-throughput sequencing technologies have opened a new era in population genetics, as they allow the assessment of complex demographic scenarios, including how gene flow and introgression affect genetic diversity and speciation [22]. In this context, our approach is to combine cytogenetic and genomic tools in an integrated way. For this, we analyzed three distinct populations of *E. erythrinus* (one of them cytogenetically investigated for the first time) and used genomic approaches to investigate how the evolution of multiple sex chromosomes together with allopatry is linked to genetic diversity and speciation. We hypothesize that the presence of multiple sex chromosomes has differentially contributed to the genetic differentiation of populations, which could have potentially accelerated speciation.

## 2. Materials and Methods

### 2.1. Specimen Sampling

**Figure 1:**
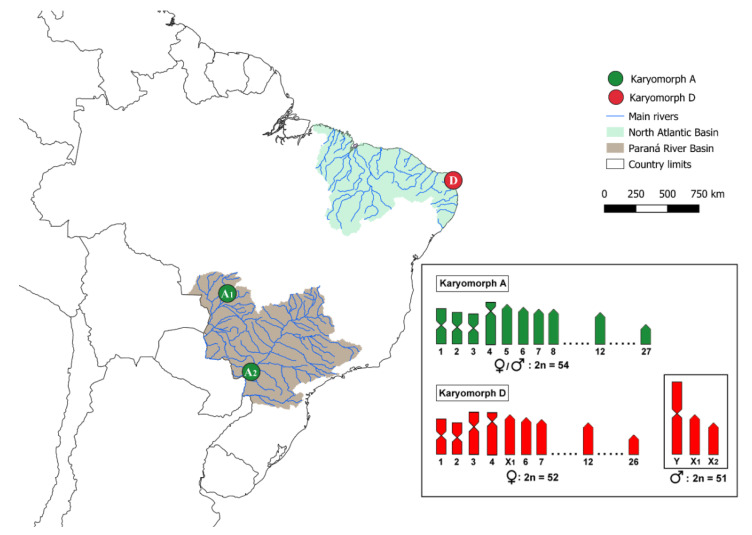
Map of South America indicating *E. erythrinus* sampling sites. The Brazilian hydrographic basins in which the samples were collected are shown; colored circles specify the sampling sites, with their corresponding karyomorphs: A, green; D, red. Sampling site codes follow those presented in Table 1. The idiograms on the right represent partial karyotypes of each karyomorph.

The collection sites, number and sex of the specimens investigated are presented in Figure 1 and Table 1. Part of the sampling (Groups A1 and D) was previously analyzed with different cytogenetic and molecular methods [7,23]. All animals were collected with proper authorization of the Brazilian environmental agency ICMBIO/SISBIO (license 48628-14) and SISGEN (A96FF09). Experiments followed the ethical conducts approved by the Ethics Committee on Animal Experimentation of the Universidade Federal de São Carlos (process number CEUA 1853260315)

### 2.2. Chromosome Preparations and Fluorescence In Situ Hybridization (FISH) for rDNA Mapping

Here we present the first chromosomal data for population A2. Mitotic chromosomes were obtained by the protocol previously described [24]. The 5S rDNA probe included the 5S rDNA coding region, with 120 base pairs (bp), and the 200 bp-long non-transcribed spacer (NTS) [25]. The 18S rDNA probes were obtained by PCR using primers previously described [26]. The 18S and 5S rDNA probes were labeled directly with a Nick Translation Labeling Kit (Jena Bioscience, Jena, Germany); 18S rDNA was labeled with Atto488 (green fluorescence) and 5S rDNA with Atto550 (red fluorescence), according to the manufacturer’s manual. FISH was conducted under high stringency conditions, as described [27].

### 2.3. Comparative Genomic Hybridization (CGH)

Comparative genomic hybridization (CGH) experiments were performed as described [28] with adaptations of probe/C_0_t-1 DNA ratio based on previous studies [28,29,30]. Male genomic probes from all populations were co-hybridized to the male chromosomes of populations A1 and D separately. For this experimental scheme, male-derived whole-genome DNA (gDNA) of all analyzed populations was extracted from liver tissue by the standard phenol–chloroform–isoamyl alcohol method [31] and labelled with ATTO550-dUTP and AF488-dUTP using a Nick Translation Mix Kit (Jena Bioscience, Jena, Germany). To block the excess of shared repetitive sequences, an unlabelled C_0_t-1 DNA, obtained from each population according to a previous study [32], was included in the final probe mixture. For each slide, a male genomic probe (500 ng each) and 15 µg of female-derived C_0_t-1 DNA from the respective population were co-precipitated in 96% ethanol, and the dry pellets were resuspended in 20 µL of hybridization buffer containing 50% formamide, 2 × SSC, 10% dextran sulphate and Denhardt’s buffer (pH 7.0). FISH was conducted under high stringency conditions, as described in [27].

### 2.4. Microscopy and Image Processing

To confirm the diploid number, karyotype structure and hybridization results, at least 30 metaphase spreads were analyzed per individual. The microscopy images were captured using an Olympus BX50 epifluorescence microscope (Olympus Corporation, Ishikawa, Japan) coupled with a CoolSNAP camera, and the images were processed using Image-Pro Plus 4.1 Software (Media Cybernetics, Silver Spring, MD, USA). Final images were optimized and arranged using Adobe Photoshop, version CC 2020. Chromosomes were classified as metacentric (m), submetacentric (sm), subtelocentric (st) or acrocentric (a), according to their arm ratios [33]. As the male and female results showed no differences, only male metaphases are presented. Idiograms were constructed using Adobe Photoshop, version CC 2020, based on previous and actual study.

### 2.5. Sequencing and Filtering

The DArTseq sequencing procedures were performed using liver tissues that were shipped to Diversity Arrays Technology Pty Ltd. (Canberra, Australia). This method involves a complexity reduction in which the DNA is treated with PstI and SbfI enzymes before sequencing, which might enrich for sequences in lightly methylated regions [34]. Sequencing was carried out on an Illumina HiSeq 2500 platform; a single-end sequencing was performed yielding 77 bp reads. This sequencing technique was implemented in several recent papers investigating fish population genomics [26,35,36]. The raw data processing and all subsequent steps to obtain the final datasets were executed using pyRAD v3.0.66 [37]. The minimal depth of coverage to form a cluster was set to 6; the clustering threshold implemented was 0.88. In the last alignment step, the minimal sample coverage for a locus was set to 18, therefore only data present in all individuals were maintained. The sequencing adapters were trimmed and sequences with more than five low-quality bases were removed (Q < 20). Subsequently, reads were aligned, and consensus sequences were obtained for each sample, which were used to define the reference base at each position in each alignment. We obtained loci that were present in all sampled individuals and generated two different datasets also in pyRAD v3.0.66 [37] for further analysis: (1) a phased sequence file for each loci that was implemented only in the genetic diversity and differentiation analyses based in the “.alleles format” and (2) a matrix of polymorphic single nucleotide polymorphisms (SNPs), with a single SNP per locus to prevent the presence of closely linked SNPs based in the “.usnps.geno” output. The SNP matrix was coded as follows: 0 for homozygotes for the reference base, 1 for heterozygotes and 2 for the alternate base homozygotes. Indels were not considered, and this second input was used in the remaining analyses.

### 2.6. Detection of Markers Putatively under Selection

A BayeScan analysis was carried out to test for markers with uncommon levels of inter-population differentiation (low or high), which might indicate locally adaptive alleles at the sequenced loci [38]. We performed runs with a prior odds value of 100, a thinning of 10 and 5000 MCMC chains. All loci with false discovery rate (FDR) values less than 0.01 were classified as possibly under selection.

### 2.7. Genetic Diversity

Genetic diversity indexes were obtained with the software DnaSP v. 6.12.03 [39]. We calculated thehaplotype diversity (*H_d_*), Tajima’s D (*D*) and two measures of nucleotide diversity per site, π and Watterson’s theta (*θ_W_*) averaged per phased loci from dataset 1. We also estimated the nucleotide divergence between pairs of samples from different sites using the average number of nucleotide substitutions per site (*D_XY_*) and the net divergence, corrected for the variation within analyzed samples (*D_A_*).

### 2.8. Population Structure Analysis

We assessed population structure with a principal coordinate analysis (PCoA) to visualize genetic diversity distribution in each karyomorph. Then, we implemented the fastStructure Bayesian approach [40]. This method is analogous to the widely used software Structure [41] but optimized for larger datasets. All results were compressed and uploaded to Clumpak [42] for a graphical depiction of the outputs. Furthermore, we carried out the spatially explicit approach in the package ConStruct [43], as freshwater fish are one of the most susceptible groups to the confounding effects of isolation by distance in population structure [44]. We executed the analysis in Rstudio with spatial parameters activated and pairwise distances compiled based on sampling site coordinates. We performed the analysis with K values ranging from 1 to 4, with a total of 1,000,000 iterations for each K value. After the runs were completed, we analyzed the contribution of each K layer to the population structure of *E. erythrinus*, as measured by their impact on the model’s covariance. Therefore, when a given K value did not contribute anymore to the result, the previous K was selected [45].

## 3. Results

### 3.1. Karyotypes and rDNA Mapping

**Figure 2:**
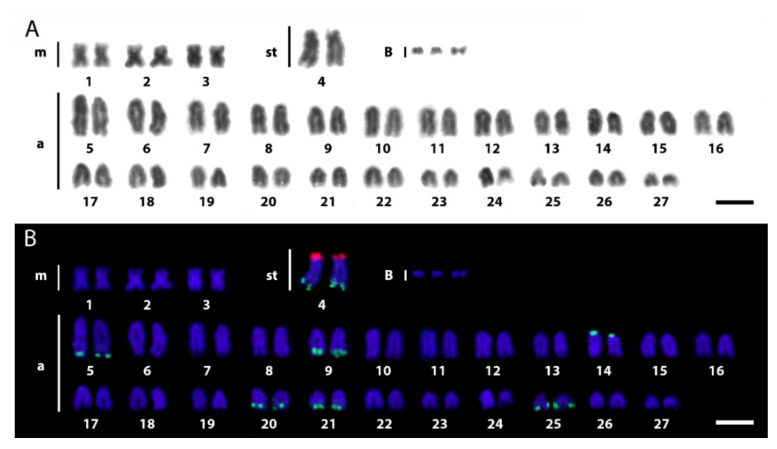
Karyotypes of *E. erythrinus* karyomorph A2 analyzed by conventional Giemsa staining (**A**) and double FISH with 18S (green) and 5S rDNAs (red) as probes (**B**). Bar = 5 µm.

The karyotype of the newly analyzed A2 population displayed the general features present on karyomorph A from the other previously assessed *E. erythrinus* populations, i.e., many acrocentric and few bi-armed chromosomes. The karyotype presented 2*n* = 54 (6m + 2st + 46a), without differentiated sex chromosomes. In addition, up to three supernumerary B chromosomes were observed, which usually appeared like double-minute chromosomes and stained less than the standard chromosomes (Figure 2). From the 30 analyzed metaphases, 20, 08 and 02 presented 3, 2 and 1 B chromosomes, respectively. Similar karyotypes, FISH and CGH patterns were observed for all metaphase spreads analyzed in all karyomorphs.

**Figure 3:**
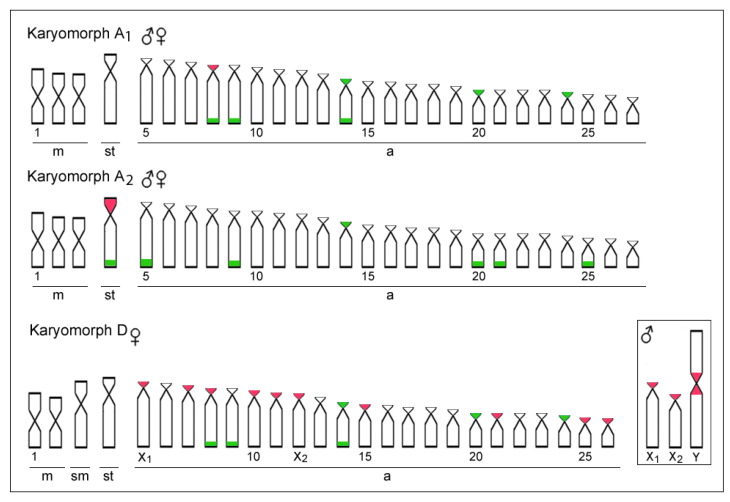
Representative idiograms of *Erythrinus erythrinus* karyomorphs A1, A2, and D highlighting the chromosomal distribution of the 18S rDNA (green) and 5S rDNA sequences (red). The sex chromosomes are boxed.

18S rDNA sites were mapped in the terminal region of the long arms of five acrocentric pairs (pairs 5, 9, 20, 21 and 25) and the subtelocentric pair 4, in addition to one chromosome pair with centromeric sites (pair 14). In turn, the only subtelocentric pair was observed with 5S rDNA sites on the telomeric region of the short arms. This same chromosome pair also bears 18S rDNA sites on its long arms, thus evidencing an unusual syntenic condition concerning these two classes of ribosomal DNAs (Figure 2 and Figure 3).

### 3.2. Comparative Genomic Hybridization

**Figure 4:**
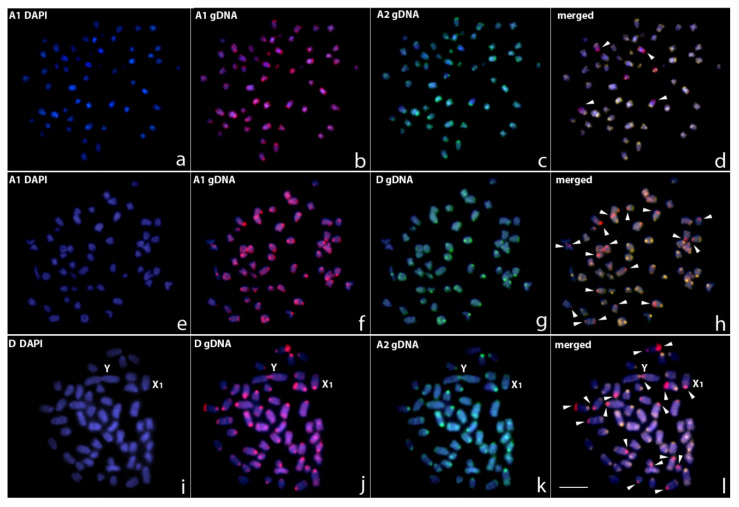
Mitotic chromosome spreads of *E. erythrinus* males after CGH—interkaryomorph comparison. (**a**–**d**) Male-derived genomic probe from karyomorph A1 (**b**) and A2 (**c**) mapped against male chromosomes of karyomorph A1. (**e**–**h**) Male-derived genomic probe from karyomorph A1 (**f**) and D (**g**) mapped against male chromosomes of karyomorph A1. (**i**–**l**) Male-derived genomic probe from karyomorph D (**j**) and A2 (**k**) mapped against male chromosomes of karyomorph D. First column (**a**,**e**,**i**): DAPI images (blue); second and third columns (**b**,**c**,**f**,**g**,**j**,**k**): hybridization pattern using the male-derived probe (red) of each analyzed karyomorph; fourth column (**d**,**h**,**l**): merged images of both genomic probes and DAPI staining. The common genomic regions of both compared karyomorphs are depicted in yellow and discrepant regions are indicated by arrowheads in the merged images (**d**,**h**,**l**). Bar = 10 μm.

The set of our CGH experiments aimed to compare genomes among the different studied populations. The intra-karyomorph comparative hybridization of gDNA probes from A1 and A2 individuals produced essentially many overlapping signals, which highlights their genomic similarities (with a stronger binding preferentially in terminal or pericentromeric heterochromatic regions) and suggests significant sequence homology, with some of them probably related to major rDNA sites. Some population-specific signals observed indicate some over-represented signals, probably linked to a higher copy number of repetitive sequences in some chromosomal regions (Figure 4a–d). On the other hand, the intra-karyomorph comparative hybridization between A1 and A2 populations with karyomorph D produced several population-specific signals with only a limited number of overlapping signals showing a higher level of genomic differentiation (Figure 4e–l).

### 3.3. Sequencing, Data Preparation and Detection of Selection Markers

Sequencing generated around 2 million reads for each sample. After trimming and filtering steps, we obtained 14,467 phased loci with a minimum coverage of six present in all individuals, which we refer hereafter as dataset 1. Then, we created dataset 2 by maintaining only one biallelic SNP per sequence locus to avoid tightly linked SNPs, which resulted in 8597 polymorphic SNPs. Bayescan did not consider any locus as potentially under selection, therefore no marker was removed from subsequent analysis.

### 3.4. Population Structure

**Figure 5:**
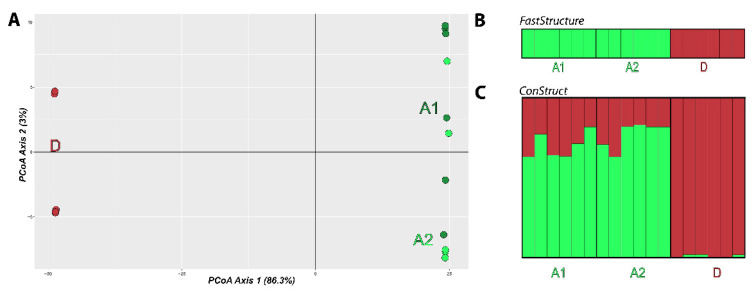
(**A**) Principal coordinate analysis. Individuals are represented in different colors as follows: A1 in dark green; A2 in light green; D in red. (**B**) FastStructure result for K = 2. Each vertical bar represents an individual; karyomorphs and sampling locations are indicated below; bar colors indicate the population to which each individual was classified. (**C**) ConStruct result for K = 2. Each vertical bar represents an individual; karyomorphs and sampling locations are indicated below.

The PCoA indicates a clear separation between karyomorphs A and D. Karyomorph D formed two minor groups that are closely related, and karyomorphs A1 and A2 formed one major cluster each, with some individuals scattered between these two clusters (Figure 5A).

The non-geographic Bayesian approach fastStructure was congruent with the PcoA result and pointed out K = 2 as the value that maximizes likelihood and best explains the data. The analysis clustered A1 and A2 as a single population and karyomorph D as a second population (Figure 5B). The spatially explicit ConStruct approach presented a result that is also congruent with both the PcoA and fastStructure results. Individuals from karyomorph A are highly similar, while those from karyomorph D formed a distinct group (Figure 5C).

### 3.5. Genetic Diversity

Overall, all sampled groups presented low diversity values. Values of nucleotide diversity (π) and Watterson theta (*θ_W_*) were higher in the A1 locality, while the lowest values were present in D. All sampled groups presented negative Tajima’s D values, with the lowest in A1, followed by A2 and then D. Values of genetic differentiation, *D_XY_* and *D_A_*, were approximately ten times lower within karyomorph A when values between karyomorphs A and D were contrasted (Table 2 and Table 3).

## 4. Discussion

### 4.1. Chromosomal Diversity among E. erythrinus Populations

A general conservative chromosomal macrostructure of *E. erythrinus* karyomorphs is not maintained when comparing intrachromosomal features of the studied subpopulations. This is evidenced by the differential distributions of the repetitive DNA fraction among them (Figure 3 and Figure 4). The new chromosomal features of the *E. erythrinus* A2 population agree with those found in other populations belonging to karyomorph A, i.e., 2*n* = 54, karyotypes dominated by acrocentric chromosomes and multiple rDNA sites (Figure 2). While the majority of erythrinid fish tend to maintain the karyotypes dominated by bi-armed chromosomes, *E. erythrinus* presents exceptions to this general rule [3,7,46] (present study).

Ribosomal DNA mapping has been extensively used in many modern cytogenetic investigations (reviewed in [47,48]), constituting a valuable marker for cytotaxonomy [49,50,51,52]. In fish, copy number variation in rDNAs is frequently observed, since their gene regulation processes appear to be more relaxed in comparison with those of higher vertebrates [53]. For *E. erythrinus*, the 5S rDNA is probably associated with the transposon Rex3, which can explain the high dispersion of this sequence in their chromosomes [7]. The karyomorph A1 displays signals in the short arms of the 8th pair, A2 in the p arms of the large subtelocentric (pair 4). Additionally, karyomorph D presents ten chromosomes with 5S sequences, mostly in short arms (Figure 3). Karyomorphs A1 and D present a very similar pattern for the hybridization of the 18S rDNA probe. Such a similar pattern is not found in the C karyomorph [23].

A substantial number of fish species present ribosomal DNA sites mapped on heteromorphic sex chromosomes [48], which can be explained by the presence of evolutionary breakpoint regions (EBRs), in several fish groups [50,54,55,56]. This feature is evident in karyomorph D, where 5S rDNA sequences can be identified in both X_1_ and X_2_ chromosomes and in the neo-Y chromosome [7] (Figure 3). On the other hand, the 18S rDNA presents a more conserved pattern of distribution on chromosomes for *E. erythrinus*, since both karyomorphs A1 and D reveal the same hybridization results for this probe. However, karyomorph A2 (herein described) presents six chromosomes mapped with the 18S rDNA probe (pairs 5, 9, 20, 21 and 25 in the terminal region of the q arms, and pair 14 in the p arms). Differences in the distribution of the 18S rDNA among populations have already been described for fish of the same family, as in the case of *Hoplias malabaricus* (reviewed [28]). An investigation of three distinct fish orders (Characiformes, Siluriformes and Perciformes) also revealed an association between the quality of the aquatic environment and the dispersion of rDNA sequences [57].

The set of our CGH experiments aimed to compare genomes among the different studied populations. Such comparisons have been found to be very effective in identifying hidden biodiversity in fish (reviewed in [28]) and in this study they have unlocked novel views and widened our understanding of the ongoing processes of inter-karyomorph genome differentiation. The degree of genomic divergence agrees with their chromosomal features, since the karyomorphs exhibited an advanced stage of sequence divergence (Figure 4). Although distinct populations of karyomorph A possess a conserved karyotype macrostructure, intra-karyomorph genomic divergences were also observed. These findings are indicative of ongoing evolutionary processes that could be responsible for driving the divergence of and possibly also speciation within *E. erythrinus* karyomorphs. Among fish, species with conserved karyotype macrostructures are also reported to have a variable pattern of genomic organization, as observed for *Lebiasina* [58], for the giant trahiras [36] and for some Osteoglossiformes species [59]. Differentiated sex chromosomes also seem to play an important role in the divergence of repetitive genomic regions, since species that show such characteristics also have distinct patterns of hybridization in CGH, as observed in armored catfish [60], trahiras [61], pencilfish [62] and characiforms with a stable ZZ/ZW sex chromosome system [63]. In fact, the role of sex chromosomes in genomic divergence among related species is not well established. Our cytogenetic and genomic results point to a higher genomic divergence in the population of *E. erythrinus* that presents a differentiated sex chromosome (karyomorph D). This is in accordance with previous findings that the emergence of a new sex-linked locus or the translocation of an existing one is one of the fundamental steps in fish sex chromosome differentiation [20,64]. Such turnover can be responsible for the higher genomic divergence pattern observed in CGH (Figure 4). Moreover, such diversity is also reflected by the higher genetic diversity observed for populations harboring such multiple systems, as discussed below.

### 4.2. Genetic Diversity and Population Structure

This is the first study that has investigated genetic diversity in *E. erythrinus*, therefore it is only possible to compare our results with those from other species. The mean value of nucleotide diversity (π) for *E. eryhtrinus* was 0.00077, while studies in other fish species gave higher values, as in Nassau Grouper (0.3566 with RAD-Seq data) [65]. Conversely, a similar mean value of π was obtained for *H. malabaricus* with DArTSeq data (0.001172) [66]. These lower diversity estimates when compared to RAD-seq (though similar to DArTSeq) were expected due to the characteristics of the sequencing method implemented in our study, since the DArTSeq procedure may enrich regions that are potentially being expressed and therefore may be under selective restraints [34]. It is important to note, however, that Bayescan analysis did not find any markers potentially under selection.

Moreover, our results indicate that karyomorphs A and D are already highly differentiated in both chromosomal and genomic features. When considering the two differentiation metrics implemented here (*D_XY_* and *D_A_* values), it is evident that groups A1 and A2 are very similar, while karyomorph D is much more different from these groups, with values at least ten times higher. All groups showed negative Tajima’s D values, which measures the difference between the mean number of pairwise differences and the number of segregating sites. There are three main hypotheses for this result: (i) purifying selection is removing variation from these groups and maintaining their genetic diversity stably (which can also be a consequence of DArTSeq methodology); (ii) genetic drift may be influencing the genetic diversity of these groups, mainly because they are sedentary which may favor the occurrence of inbreeding [3]; (iii) the population size was low, and a population size expansion occurred recently.

The current results and the divergence patterns observed are probably explained by two main factors. The first is that samples from the same karyomorph are expected to be more similar, although that is not a general rule. The second factor is the distance between these groups and, consequently, the probable time since the isolation event occurred. All karyomorph A groups are found in the Paraná River Basin, although they are in very distant regions. Additionally, *E. erythrinus* presents sedentary behavior and is not a migrant species. Taking all these facts into account, even though the two sampling localities are in the same geographic basin, they are somewhat isolated. When comparing karyomorphs A and D, we have a different scenario, since they are geographically distant from each other and are also different in terms of the presence of sex chromosomes, which could accelerate the differentiation of these populations and potentially lead to speciation.

The population structure analysis (Figure 5) indicated that there are two main clusters of individuals separated according to their karyomorphs. As can be seen in the PCoA, karyomorphs A and D are very distant on the horizontal axis that comprises 86.3% of the variation. Looking closely at each karyomorph on the vertical axis, which comprises 3% of the variation, there are two distinct groups of individuals inside karyomorph D. In addition, inside karyomorph A there is a small difference between the A1 and A2 groups, but many individuals are distributed along the *Y*-axis. This indicates that there is a higher diversity within and a low difference between the localities for karyomorph A. The Bayesian non-spatial population structure approach fastStructure (Figure 5B), PCoA results and the geographical approach ConStruct (Figure 5C) are all congruent. According to these analyses, there are two main populations, with karyomorph A represented by one of these populations and karyomorph D represented by the other. Additionally, it is possible to see in Figure 5C that the individuals from the A1 and A2 groups present higher diversity, with a more diverse genetic composition. These results are also in accordance with the cytogenetic results, which also highlighted such inner chromosomal variability. In contrast, karyomorph D individuals present an almost uniform genetic composition.

### 4.3. Multiple Sex Chromosome Systems and the Speciation Process

The results indicate that the presence of the multiple sex chromosome system in karyomorph D may have contributed to the fixation or the increase of previously achieved genetic differences, which may have stimulated reproductive isolation and consequent speciation in the evolutionary process of *Erythrinus*.

Among erythrinids, it has been recently highlighted in another study that the presence of a multiple sex chromosome system may be associated with the speciation process [66]. *Hoplias malabaricus* is a species that is also considered a “species complex”. It is divided into seven distinct karyomorphs that also vary in their 2*n*, karyotypic structure and in the presence of simple and multiple sex chromosome systems [67], coupling genomic and cytogenetic analysis comprising several populations from different karyomorphs has reinforced that *H. malabaricus* is probably a species complex rather than a single species and highlighted the potential role of the neo-sex chromosome system favoring the speciation process [66]. In *H. malabaricus*, individuals from karyomorphs A (homomorphic sex chromosomes) and D (multiple ♀X_1_X_1_X_2_X_2_/♂X_1_X_2_Y system) were collected in sintopy (the same location) and presented high genetic differentiation. According to values of *D_XY_*, the genetic differentiation between syntopic individuals from karyomorphs A and D was equivalent to the genetic differentiation of long-distance allopatric populations of karyomorph A. Other studies indicated that the emergence of neo-sex chromosomes could have contributed to the increase in genetic diversity (and potentially to speciation), as demonstrated in sticklebacks [68] and in the plant *Rumex hastatulus* [69]. In all the above-mentioned cases, events of sex chromosome turnover led to the emergence of neo-sex chromosomes with the potential to fix genetic variation, mainly due to the rapid evolution of these chromosomes [69].

## 5. Conclusions

Our results indicated the presence of high genetic differentiation among populations of the species complex *E. erythrinus* both from cytogenetic and genetic points of view. Even though the genetic diversity indexes were globally low, karyomorphs A and D are highly differentiated genetically. In addition, three main hypotheses for the differentiation found can be proposed. (1) Long-distance allopatry could have been the main agent of genetic differentiation between these karyomorphs. (2) The emergence of neo-sex chromosomes may have been the main factor that promoted this genetic differentiation. (3) Both long distance allopatry and the emergence of the neo-sex chromosomes on karyomorph D may have contributed to the fixation and accumulation of divergences. Hypothesis 3 is our main hypothesis. In summary, our data provided an additional layer of evidence for the status of the taxon *E. erythrinus* and corroborated previous studies, supporting the conclusion that it is probably a species complex with an unresolved taxonomy. Future studies using high-throughput sequencing focusing on sex determination in this complex will help to highlight the possible link of sex chromosomes with the speciation process in this group.

## Figures and Tables

**Table 1:** Summary of individuals of *E. erythrinus* analyzed, karyomorphs, diploid chromosome numbers (2*n*), sex chromosome systems, group identification codes (indicating the sampling sites and also the karyomorph name), numbers of individuals cytogenetically analyzed from each site (Cito_N), number of individuals sequenced (DArT) and references.

Karyomorph	2*n*	Sex System	Code	Sampling Site	Latitude/Longitude	Cito_N	DArT	Reference
A	♀♂ 54	Homomorphic	A1	Cuiabá River (MT)	−16.1713/−55.9573	14♂ 11♀	6	[23]
A	♀♂ 54	Homomorphic	A2	Paraná River (PR)	−23.3774/−53.7805	09♂ 07♀	5	Present work
D	♀52/♂51	♀X_1_X_1_X_2_X_2_ ♂X_1_X_2_Y	D	Sangue Stream (RN)	−5.7770/−35.2092	10♂ 09♀	7	[7]

Abbreviations: MT = Mato Grosso, PR = Paraná, RN = Rio Grande do Norte Brazilian States.

**Table 2:** Calculated average genetic diversity per loci by sampling site. Sample sizes are shown, as well as haplotype diversity (*H_d_*), nucleotide diversity (π), Watterson theta per site (*θ_W_*) and Tajima’s D (*D*).

Code	Sample Size	*H_d_*	π	*θ_W_*	*D*
A1	6	0.05290	0.00102	0.00125	−0.48480
A2	6	0.04306	0.00085	0.00093	−0.25292
D1	12	0.02115	0.00044	0.00047	−0.19581

**Table 3:** Pairwise DXY per sampling site is represented in the upper diagonal, pairwise DA in the lower diagonal.

	A1	A2	D
A1		0.00102	0.01084
A2	0.00012		0.01082
D	0.01014	0.01022	

## Data Availability

The data presented in this study are available at https://github.com/Fernando-H-S-Souza/Biology-Manuscript-biology-11-00315-Datasets-1-and-2, accessed on 1 February 2022.
